# Higher serum S100B and BDNF levels are correlated with a lower pressure-pain threshold in fibromyalgia

**DOI:** 10.1186/1744-8069-10-46

**Published:** 2014-07-08

**Authors:** Simone Azevedo Zanette, Jairo Alberto Dussan-Sarria, Andressa Souza, Alicia Deitos, Iraci Lucena Silva Torres, Wolnei Caumo

**Affiliations:** 1 Physical Medicine and Rehabilitation Service at Hospital de Clínicas de Porto Alegre (HCPA), Universidade Federal do Rio Grande do Sul (UFRGS), Porto Alegre, Brazil; 2 Post Graduate Program in Medical Sciences, UFRGS, Porto Alegre, Brazil; 3 Laboratory of Pain & Neuromodulation, HCPA/UFRGS, Porto Alegre, Brazil; 4 Pain and Palliative Care Service at HCPA/UFRGS, Porto Alegre, Brazil; 5 Universitary Center Unilasalle, Canoas, Brazil; 6 Pharmacology Department, Instituto de Ciências Básicas da Saúde, UFRGS, Porto Alegre, Brazil

**Keywords:** Fibromyalgia, S100B, BDNF, Pain-pressure threshold, Central sensitization

## Abstract

**Background:**

Fibromyalgia (FM) is conceptualized as a central sensitization (CS) condition, that presents high serum brain-derived neurotrophic factor (BDNF) and neuroglia activation. Although the S100B protein regulates neuroglia functions, it has been traditionally used as a proxy of central nervous system damage. However, neither BDNF nor S100B association with the clinical picture of FM has been elucidated. To explore their association with the pressure-pain threshold (PPT) in FM, we performed a cross-sectional study, including 56 females with confirmed FM aged 18–65 years. Linear regression models were used to adjust for potential confounding factors between serum BDNF, S100B and PPT.

**Results:**

Serum BDNF and S100B were correlated (Spearman’s Rho = 0.29). Serum BDNF (*log*) and S100B (*log*) were correlated with the PPT (*log*) (Partial η^2^ = 0.129, *P* = 0.012 for the BDNF (*log*), and Partial η^2^ = 0.105, *P* = 0.025 for the S100B (*log*)). Serum BDNF (*log*) was inversely associated with PPT (*log*) (β = -1.01, SE = 0.41), age (β = -0.02, SE = 0.15) and obsessive compulsive disorder (β = -0.36, SE = 0.15), while serum S100B (*log*) was inversely associated with PPT (*log*) (β = -1.38, SE = 0.50), only.

**Conclusions:**

Both neuroglia key mediators in the CS process were inversely correlated with the PPT. Serum assessment of BDNF and S100B deserve further study to determine its potential as a proxy for the CS spectrum in FM.

## Background

Fibromyalgia (FM) is estimated to affect between 1.6% [1] and 6.4% [2] of the population, and is characterized by widespread chronic pain accompanied by fatigue, non-satisfying sleep and cognitive symptoms [3]. Although its pathophysiology is not fully understood, abnormalities in pain processing [4] related to central sensitization (CS) and a reduced descending pain modulation with sensory amplification [5,6] have been recognized as components of FM. In the CS model, pain hypersensitivity and enhanced receptive field characteristics of the disease [7] may be explained as a consequence of increased neuronal membrane excitability, synaptic facilitation and nociceptive pathway disinhibition mediated at the molecular level by the modification of receptor kinetics (*e.g.,* N-methyl-D-aspartate (NMDA) and α-amino-3-hydroxy-5-methyl-4-isoxazolepropionic acid (AMPA)) [8] and at the cellular level by the interaction of both neurons and microglia interchanging neurotransmitters and inflammatory cytokines (*e.g.,* substance P, tumor necrosis factor alpha, and brain-derived neurotrophic factor (BDNF)), which results in enhanced neuronal and nociceptive pathway functions [8-10].

BDNF is a neurotrophic factor that is widely distributed in the central nervous system (CNS) and is capable of strengthening excitatory (glutamatergic) synapses as well as weakening inhibitory (GABAergic) synapses [11]. In the context of CS, microglial cells activated by astrocytes can also release BDNF, which reduces the expression of the Cl^-^-cotransporter K^+^-Cl^-^ exporter (KCC2) in the dorsal horn. This results in the accumulation of intracellular Cl^-^, which further limits the GABAergic inhibitory effect on these nociceptors, thereby promoting disinhibition [8]. BDNF produced in the CNS is transported through the blood–brain barrier via saturable systems [12-14], and contributes to 70-80% of circulating BDNF [15]. Although circulating levels of BDNF have been found to be elevated in FM compared with controls, its association with the patients’ clinical complaints remains elusive.

Despite known role of astrocytes and microglia in the CS [16], surrogates of their activity other than BDNF have been scarcely explored in FM patients. S100B is a Ca^2+^-binding protein that, at higher concentrations, upregulates Interleukin-1β and Tumor Necrosis Factor α expression and activates the Nuclear Factor kappa-light-chain-enhancer of activated B cells (NF-kB) via the receptor for advanced glycation end products (RAGE) in microglia and astrocytes [17,18], all of which are involved in CS progression and maintenance [8]. Elevated S100B serum levels have been correlated with mood disorders [19] and have been studied as an outcome predictor in severe traumatic brain injury [20,21] but have not been previously explored in a chronic pain syndrome.

Thus, considering that both BDNF and S100B play relevant roles in CS and that both factors can be assessed in serum, we hypothesized that 1) serum levels of both BDNF and S100B may be associated with FM, and 2) both serum mediators could have an association with the pressure-pain threshold (PPT). To test our hypotheses, we performed a cross-sectional study of FM patients, examined their serum BDNF and S100B levels, and evaluated their clinical and algometrical characteristics.

## Results

**Figure 1:**
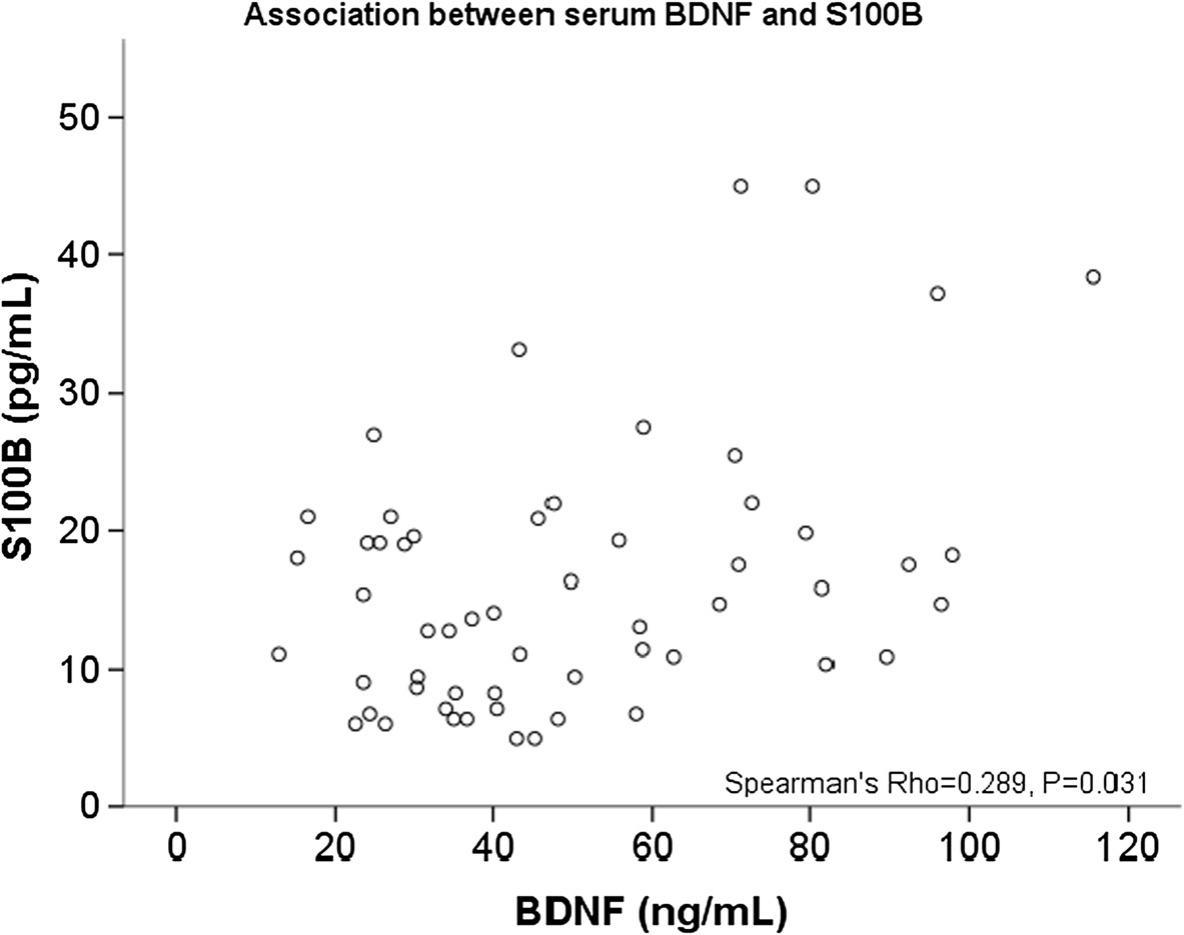
Scatter plots of serum S100B and BDNF.

Two hundred and seventy-one patients were screened, and 215 patients were excluded (205 patients did not meet the inclusion criteria, and 10 of the patients did not consent to participate). Fifty-six subjects were included in the final analysis. The demographic and clinical characteristics of the sample are described in Table 1. The assessed levels of serum BDNF and S100B are described in Table 2. Both serum BDNF and S100B showed a significant non-parametric correlation (Spearman’s Rho = 0.289, *P* = 0.031), which means that the patients with higher BDNF presented higher S100B (Figure 1).

**Table 1:** Characteristics of the sample (n = 56)

**Characteristic**	**Mean ± SD**
Age (years)	48.78 ± 7.91
Body mass index (kg/m^2^)	27.38 ± 3.89
Years of education (median (Q_25–75_))	11 (6–14)
Number of trigger points (median (Q_25–75_))	14 (13–16)
Employed (yes/no)	41/15
Smoking (yes/no)	4/52
Alcohol use (yes/no)	7/49
Psychiatric disorder according to the MINI (yes/no)*	
Major depressive episode	32/23
Major depressive episode with dysthymia	18/37
Dysthymia	10/45
Suicidal risk	11/44
Hypo-maniac episode	13/42
Panic disorder	12/43
Agoraphobia	22/33
Social phobia	11/44
Obsessive compulsive disorder	12/43
Post-traumatic stress disorder	3/52
Psychotic syndrome	1/54
Generalized anxiety disorder	28/27
Maniac-depressive disorder	2/53
Psychotropic drugs (yes/no)**	38/18
Tricyclic antidepressant (yes/no)	24/15
Selective serotonin reuptake inhibitor (yes/no)	10/28
Benzodiazepine	4/35
Other chronic disease (yes/no)	28/28
Hypertension (yes/no)	14/42
Type 2 Diabetes Mellitus (yes/no)	1/55
Asthma (yes/no)	14/42
Number of analgesic doses used per week (median (Q_25–75_))	14 (7–21)
Widespread Pain Index (WPI)	15.11 ± 2.04
Symptom Severity (SS) Scale Score	7.43 ± 1.51
Pain on the VAS	70.21 ± 14.54
Pittsburgh Sleep Quality Index	22.09 ± 7.35
Hamilton Depression Rating Scale	19.93 ± 5.81
Pain Catastrophizing Scale (B-PCS) score	30.73 ± 11.65
Hopelessness	11.38 ± 4.91
Magnification	12 (7–15)
Rumination	8.14 ± 2.42
Pressure pain threshold (kg/cm^2^)	2.09 ± 0.23
Fibromyalgia Impact Questionnaire	61.20 ± 12.82

*Patients could have none or more than one psychiatric disorder. One patient missed the interview for the Mini International Neuropsychiatric Interview (MINI) application.

**Some patients were using more than one type of drug.

**Table 2:** Serum concentration of BDNF and S100B (n = 56)

**Serum marker**	**Median (**_**Q25–75**_**)**	**Mean ± SD**
S100B (pg/mL)	14.42 (8.78 - 19.77)	16.15 ± 9.57
BDNF (ng/mL)	43.37 (30.03 - 69.99)	49.72 ± 24.84

To study the potential effect of confounders, multiple linear regressions were performed independently using the logarithmic transformation of both BDNF and S100B as dependent variables and the clinical characteristics with the potential confounding effect as covariates, including age, body mass index (BMI), years of education, employment status, smoking status, use of alcohol (yes/no), diagnosis of arterial hypertension, psychiatric diagnoses according to the MINI, frequency of use of over-the-counter analgesics, mean pain on the visual analog scale on the 0–100 mm scale (VAS_0–100_), score on the Pittsburgh Sleep Quality Index (PSQI), score on the Hamilton Depression Rating Scale (HDRS), Pain Catastrophizing Scale score adapted for the Brazilian population (B-PCS) and its dominions, the Fibromyalgia Impact Questionnaire (FIQ), the PPT (*log*), the Widespread Pain Index (WPI) and the Symptom Severity (SS) scale score.

Variables that were independently correlated with BDNF (*log*) and S100B (*log*) significant in the multiple linear regression models included age, log PPT and obsessive compulsive disorder according to the MINI. These variables were subsequently entered as independent variables in the multivariate linear regression model, where both BDNF and S100B logarithmic transformations were entered as dependent variables. The analysis showed a significant correlation between the PPT (*log*) (Wilks’ λ = 0.809, F = 5.306 *P* = 0.009, Partial η^2^ = 0.191) and obsessive compulsive disorder (Wilks’ λ = 0.864, F = 3.553 *P* = 0.037, Partial η^2^ = 0.136) (Table 3).

**Table 3:** Multivariate linear regression model of the association between serum BDNF, S100B and clinical parameters, including the pressure-pain threshold (n = 56)

**Dependent variable**	**Type III sum of squares**	**df**	**Mean square**	**F**	**P**	**Partial eta squared**
BDNF (*log*)	4.369	3	1.456	7.855	0.001	0.339
S100B (*log*)	3.098	3	1.033	3.583	0.021	0.189
**Dependent variable**		**β**	**SEM**	**t**	***P***	**Partial eta squared**
**BDNF (*log*)**						
Intercept		5.54	0.43	12.77	<0.001	0.760
PPT (*log*)		-1.01	0.41	-2.62	0.012	0.129
Age		-0.02	0.15	-2.31	0.025	0.104
Obsessive compulsive disorder		-0.36	0.15	-2.42	0.019	0.113
**S100B (*log*)**						
Intercept		3.66	0.37	9.89	<0.001	0.189
PPT (*log*)		-1.38	0.50	-2.78	0.008	0.105

**Figure 2:**
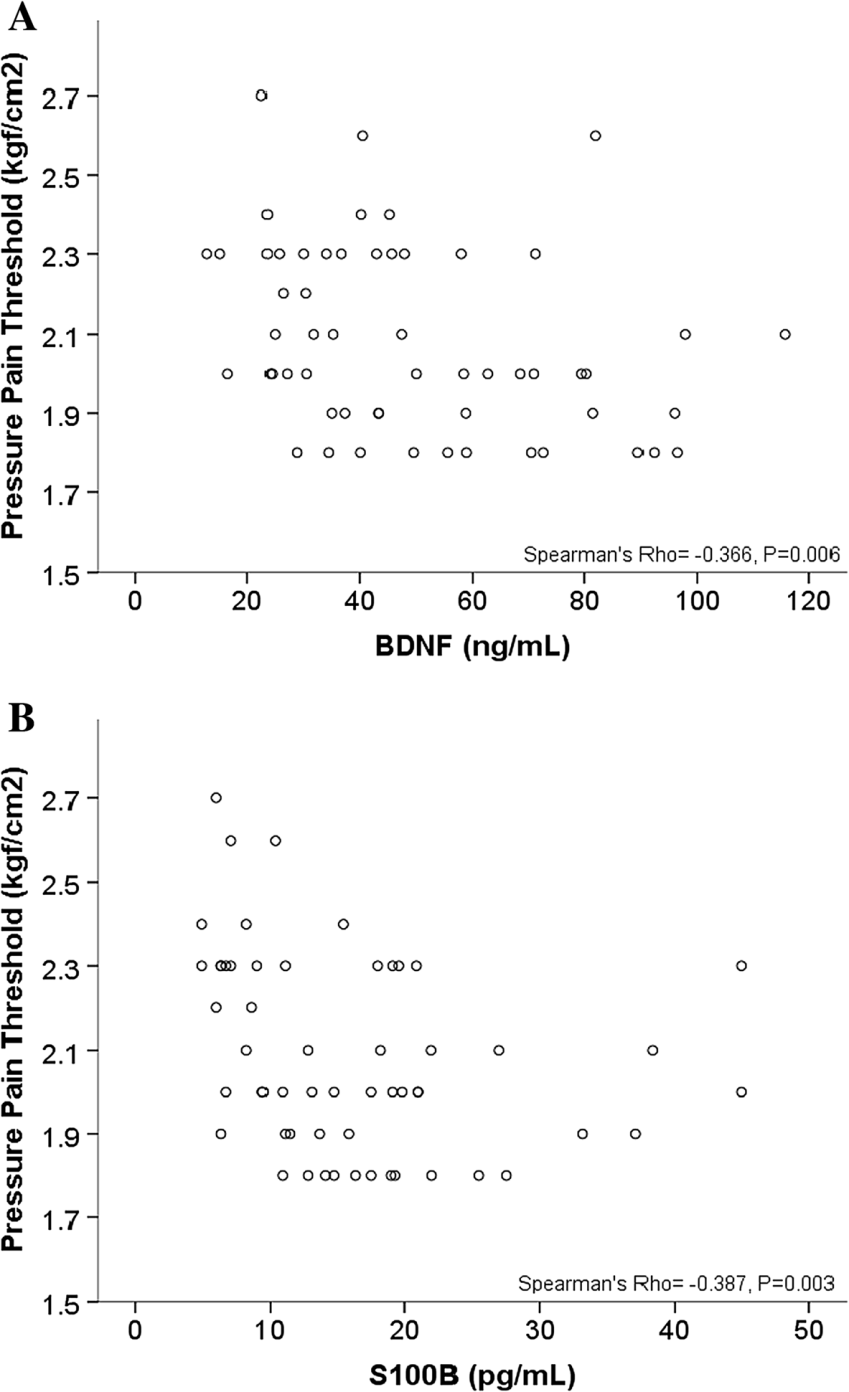
Scatter plot of the correlation between the pressure-pain threshold and serum BDNF (A) and serum S100B (B).

Estimators of the association in the multivariate model are presented in Table 3. Both serum mediators were inversely correlated with PPT (*log*). Scatter plots of the raw PPT, BDNF and S100B are presented for illustrative purposes in Figure 2, showing that an inverse non-parametric correlation between them and the PPT was also presented. Such non-parametric correlation means that patients with greater PPT had lower serum mediators. The PPT (*log*) (Cohen’s f^2^ = 0.23) had medium effect size for the BDNF (*log*), and a small effect size for the S100B (*log*) (Cohen’s f^2^ = 0.16). Although the WPI presented a non-parametric correlation with the PPT (log) (Spearman’s Rho = -0.506, *P* < 0.001) it was not correlated with BDNF (Spearman’s Rho = 0.105, *P* = 0.465) nor S100B (Spearman’s Rho = 0.260, *P* = 0.065).

## Discussion

Our results support the hypothesis of a correlation between serum BDNF and S100B in FM patients and their association with PPT. This is the first report of serum S100B levels in a human FM sample and its association with another mediator of CS, serum BDNF. Furthermore, we observed that both serum BDNF and S100B were higher in FM patients with lower PPT.

To the best of our knowledge, this is the first study to assess serum S100B in a human sample with FM. In animal models of hyperalgesia induced by chronic restraint and with unpredictable stress, serum S100B was found to be consistently elevated, although its concentration was not related to brain tissue levels [22,23]. Nevertheless, in healthy female humans, serum S100B has been correlated with its concentration in oligodendrocytes, particularly in the frontal, parietal, corpus callosum, dorsolateral prefrontal, and temporal white matter [24,25], and, to a lesser degree, in the dorsolateral prefrontal and temporal cortices [25]. Thus, the serum levels of S100B may represent white matter structural changes, which, at the cellular level, indicate that this serum mediator might be a proxy of neuroglia changes. Thus, the indirect assessment of CS is valuable because it would demonstrate the role of neuroglia in this pathological phenomenon [16]. Although astrocytic S100B has been correlated with serotonergic drug activity [19,26], our study did not assess neither adjust for serotonergic parameters or psychotropic drugs to address this possible correlation in the present sample.

BDNF had previously been studied in FM, and although its serum [27,28], plasma [29] and cerebrospinal fluid [30] levels have been consistently found to be elevated compared with controls, its association with PPT has not been previously reported in FM. A relationship between BDNF and the PPT has been previously described in healthy volunteers, and its direction is dependent on gender, where females exhibit proportional PPT and BDNF, while males demonstrate an inverse relationship [31]. Although our female FM sample had a response that contrasted to that found in healthy females and was similar to the samples of healthy males, we cannot exclude the possibility that menopause could be involved in the observed effect, due to the mean age of our sample and the lack of control for hormonal levels in our study [31]. The serum BDNF relationship with PPT may be tracked at different levels, because BDNF is widely distributed in the CNS and its binding to its high-affinity trkB receptor enhances C fiber-evoked responses, thereby activating signaling pathways in the spinothalamic track and subsequently strengthening excitatory synapses while promoting disinhibition of descending pathways [8].

Using different neuroimaging approaches (*i.e.,* magnetoencephalograpy (MEG) [32], functional magnetic resonance imaging (fMRI) [33], proton magnetic resonance spectroscopy (H-MRS) [34]), it has been shown that lower PPT is correlated with higher brain activation in FM compared to controls. Lower PPT has also been associated with higher concentrations of glutamate within the posterior insula, which supports the finding of enhanced excitatory neurotransmission in FM [34]. Furthermore, single nucleotide polymorphisms (SNPs) in the gene encoding the catechol-O-methyltransferase (COMT) (*i.e., met/met* genotype *Val158Met* SNP), which has also been postulated to contribute to the pathogenesis of FM, have been correlated with lower PPT [35]. Taken together, these findings support the validity of using PPT in FM as a proxy for these hypothesized pathophysiological mechanisms. Our results further suggest that the PPT might also represent the CS mechanisms involved in FM, because lower PPT is correlated with higher BDNF and S100B, which are mediators involved in CS. Also, the WPI was inversely correlated with the PPT, indicating that those with lower threshold tend to present greater number of painful areas.

Other clinical characteristics were also correlated with serum BDNF and S100B. The inverse correlation of age with BDNF has been previously described in healthy subjects [28] but not in FM patients. As a component of the regression modeling, the effect of other confounders on the association between PPT and serum mediators was adjusted. In more than half of our sample, a major depression disorder in the MINI was identified, thereby representing a higher prevalence than previously reported in a Canadian representative survey [36]. Nevertheless, these differences may be attributed to different diagnostic and sampling methods employed by the study. Importantly, chronic pain is strongly associated with depressive symptoms [37,38], which limits the extent to which this potential confounding factor may be controlled, because it is a component of chronic pain syndromes. Psychiatric disorders are expected to affect the association of the PPT and serum mediators studied; however, only the obsessive compulsive disorder, which is a component of the spectrum of anxiety disorders, showed an effect on serum BDNF. Although other studies have failed to demonstrate that serum BDNF levels differ between FM and controls with comparable anxiety levels [28], we cannot conclude that anxiety itself [39] or the associated antidepressant drugs [27] used had an effect on both BDNF and S100B levels. Because we only recruited females in our sample, care should be taken when extrapolating for the male population because the association of BDNF with PPT is correlated with gender in healthy subjects [31] and male FM patients present lower PPT compared to females [40].

Because CS is conceptualized as a continuum phenomenon in which FM lies at one of its extremes [41] and PPT is proportionally associated with alterations in genetic and cortical activation in the FM, our findings suggest that the serum assessment of BDNF and S100B may also be considered as a proxy of the CS spectrum in FM. A serum test related to CS and available for clinicians would provide patients and caregivers with an additional tool to improve their understanding of FM, and would help to alleviate the burden of suffering or accompanying a condition that is unfortunately socially perceived as occurring “all in the head” of patients. The cross-sectional nature of our study design does not allow for the inference of causality, and thus, future studies are required to determine how these mediators may be responsible of the reduction in PPT in FM patients, and if they may represent biomarkers for this condition. Besides that, because our study did not include healthy controls, care must be taken regarding generalization out of the FM population. Also, it is worth noting that as expected for the characteristics of the disease, plenty of patients in our sample used psychotropic drugs including antidepressants, which have been associated with modulation of the BDNF [27]. Taken together, these findings may be applicable for clinical use to define therapeutic approaches, particularly considering that PPT is a quantitative clinical tool to assess tenderness and sensitization in FM with good reproducibility and discrimination ability [22-24]. Because genetic [25] and neuroimaging studies [26-28] have consistently shown its association with changes attributed to the CS, PPT may also be considered as a powerful tool for translational purposes in FM research.

## Conclusions

This study provides additional evidence supporting the existence of a relationship between serum S100B and BDNF in FM and brings attention towards their value as clinical tools to assess CS. Taken together, these results suggest that similar to the BDNF, S100B serum levels may also be studied as a proxy of CS mechanisms in FM, raising the possibility of further exploration of its potential use as a biomarker in CS syndromes.

## Methods

The Methods and Results sections are reported according to STROBE guidelines [42]. All patients provided written informed consent prior to their participation in this study, and the protocol was approved by the Research Ethics Committee at the Hospital de Clínicas de Porto Alegre (HCPA) (Institutional Review Board IRB 0000921), according to the Declaration of Helsinki (Resolution 196/96 of National Health Council).

### Study design, settings and participants

A cross-sectional study was performed at HCPA, Brazil, between March 2011 and December 2013. Subjects were invited from the outpatient clinics of Physical Medicine & Rehabilitation at HCPA, and by newspaper advertisement. Adult female patients with a diagnosis of FM were invited to participate. We included patients with FM diagnosis confirmed by an experienced physiatrist (SAZ) according to the criteria of the American College of Rheumatology [3] and who had pain on the visual analog scale (VAS _0–100 mm_) equal to or higher than 50 mm during one week preceding the assessment. To assess pain on the VAS_0-100mm_, patients were presented a continuous 100 mm line and were asked to mark the point that best represented their mean pain during most of the time of the past week, where 0 mm represented no pain at all and 100 mm represented the worst pain ever. A psychiatric interview was also performed on patients to screen for potential disorders; however, patients were not excluded based on these diagnoses. Exclusion criteria were evidence of inflammatory rheumatic disease, autoimmune disorder, and history of substance abuse, neurological or oncologic disease, ischemic heart disease, kidney or hepatic insufficiency.

### Dependent variables

The dependent variables of interest were serum BDNF and serum S100B. Biological samples were collected early in the morning. All the materials were obtained in plastic tubes and centrifuged for 10 minutes at 4500 rpm at 4°C. Serum was frozen at -80°C until further analyses. Serum mediator concentrations were determined using BDNF (Chemicon/Millipore, catalog no. CYT306, lower detection limit of the kit = 7.8 pg/mL) and S100B (Millipore, Missouri, USA, catalog no. EZHS100B-33 K, lower detection limit of the kit = 2.7 pg/mL) enzyme-linked immunosorbent assay (ELISA) kits, according to the manufacturers’ instructions.

### Independent variables

After gathering biological samples an interview to address all the questionnaires was performed. Diagnoses of psychiatric disorders were performed according to the criteria of the psychiatrist interviewer using the MINI screening questionnaire. All of the psychological tests used in this study had been validated for the Brazilian population and were applied by a physiatrist experienced in the administration of these tests. The patients’ baseline depressive symptoms were assessed using the Hamilton Depression Rating Scale (HDRS). The pain catastrophizing scale validated for the Brazilian population (B-PCS) was a self-administered questionnaire that consisted of 13 items to assess the extent of the patients’ catastrophizing thoughts and behaviors, with a score ranging from 0 to 52 [43]. Sleep quality was assessed using the Pittsburgh Sleep Quality Index (PSQI) [44]. Demographic data and medical comorbidities were assessed using a standardized questionnaire from our laboratory. Analgesic use was assessed by the number of doses used weekly within the last 3 months. The Fibromyalgia Impact Questionnaire (FIQ) was applied because it is a disease-specific questionnaire that has been validated for use in the Brazilian population [45]. It consists of 10 domains, and the maximum possible score is 100.

To assess the pressure-pain threshold (PPT), patients were asked to differentiate the perception of pressure versus the perception of “onset of pain”. The patients were instructed to verbally report as soon as the perception of pain began. The investigator (SAZ) assessing the PPT was trained and unable to view the display of pressure intensities. An electronic algometer (J Tech Medical Industries, USA) was used. The device consisted of a 1-cm^2^ hard-rubber probe, which was applied over all of the tender points. The average values of the PPT in kgf/cm^2^ (lb/cm^2^) for three successive readings were obtained at intervals of 3–5 min and used as outcomes. The number of tender points was determined using the PPT in the classical areas previously described by the American College of Rheumatology for FM criteria [46].

### Efforts to address potential sources of bias

To reduce assessment bias, only one researcher (SAZ) was involved in all of the assessments with the exception of the MINI, which in turn was applied by one psychiatrist. The evaluator (SAZ) is a practicing physiatrist of the outpatient clinic at HCPA with vast clinical expertise, who is well trained in the application of clinical scales and PPT assessment, as well as in the care of chronic pain patients. The algometer used (J Tech Medical Industries, USA) was calibrated and was acquired from a recognized provider in Brazil.

### Sample size

Considering type I and II errors of 0.05 and 0.20, respectively, and anticipating an effect size (*f*^2^) of 0.25 for multiple regression analysis, which allows for four predictors [47], a sample size of 53 patients was estimated. A sample of 56 patients was determined to account for unexpected factors, which would decrease the study power and increase the increased variability of our sample or missing data.

### Statistical methods

Conventional central tendency statistics were used to summarize and present the main features of the sample. Distribution of the variables was tested using the Shapiro-Wilk test. Non-normally distributed variables were subjected to logarithmic transformation. After confirming the corresponding assumptions, linear regression analysis with forward selection controlling for co-linearity [48] and autocorrelation (Durbin-Watson test) was performed to identify potential confounders in the association between the main independent variables of interest (i.e., clinical scales and PPT) and serum markers (i.e., BDNF and S100B). The covariates included in the model were those that presented a correlation coefficient equal to or higher than 0.3 with a P-value <0.15, including age, body mass index, years of education, employment status, smoking status, alcohol use, diagnosis of arterial hypertension, presence of psychiatric diagnosis according to the Mini International Neuropsychiatric Interview (MINI), the Widespread Pain Index (WPI), the Symptom Severity (SS) scale score according to the ACR 2010 FM diagnostic criteria, and the number of analgesic doses used per week. Each MINI potential diagnosis was entered in the model as a dichotomous variable, where having a diagnosis (*i.e.,* major depression, major depression with dysthymia, dysthyma, suicidal risk, hypo-maniac episode, panic disorder, agoraphobia, social phobia, obsessive compulsive disorder, post-traumatic stress disorder, alcohol dependence or abuse, non-alcoholic substance abuse, psychotic syndrome, nervous anorexia, nervous bulimia, anti-social personality disorder, or maniac-depressive disorder) was coded as 1 or 0 otherwise. Only covariates (PPT, age, and obsessive compulsive disorder) that were retained in each model were included in the final multivariate linear regression model with both serum markers as dependent variables (*i.e.* BDNF and S100B). Cohen’s *f*^2^ effect size was calculated using an effect size calculator for multiple regressions given the values of R^2^[47]. The alpha level was set at 0.05. The data were analyzed using SPSS version 18.0 (SPSS, Chicago, IL).

## Abbreviations

UFRGS: Universidade Federal do Rio Grande do Sul; HCPA: Hospital de Clínicas de Porto Alegre; FM: Fibromyalgia; CS: Central Sensitization; BDNF: Brain-Derived Neurotrophic Factor; PPT: Pressure-Pain Threshold; NMDA: N-Methyl-D-Aspartate; AMPA: α-Amino-3-hydroxy-5-methyl-4-isoxazolepropionic acid; CNS: Central Nervous System; KCC2: Chloride-cotransporter Potassium and Chloride exporter; GABA: Gamma-Aminobutyric Acid; RAGE: Receptor for advanced glycation end products; BMI: Body mass index; MINI: Mini International Neuropsychiatric Interview; PSQI: Pittsburgh Sleep Quality Index; HDRS: Hamilton Depression Rating Scale; B-PCS: Pain Catastrophizing Scale validated for the Brazilian population; FIQ: Fibromyalgia Impact Questionnaire; ELISA: Enzyme-linked immunosorbent assay.

## Competing interests

The authors declare that there are no financial or other relationships that might lead to conflicts of interest for any of the following arrangements: financial relationship to the work; employees of a company; consultants for a company; stockholders of the company; members of a speaker’s bureau or any other financial form.

## Authors’ contributions

WC, ILST and SAZ conceived and designed the study. SAZ, AS and AD acquired data. JAD-S, ILST and WC analyzed and interpreted the data. WC, SAZ and JAD-S drafted the manuscript. All of authors have read and approved the final manuscript.
